# Development and internal validation of a parsimonious model for neurological deterioration at 48 h after endovascular thrombectomy

**DOI:** 10.3389/fneur.2026.1888204

**Published:** 2026-10-16

**Authors:** Guoyong Ren, Chenyang Zhao, Yaxuan Sun

**Affiliations:** Department of Neurology, Shanxi Provincial People's Hospital, Shanxi Medical University, Taiyuan, China

**Keywords:** calibration, decision curve analysis, endovascular thrombectomy, internal validation, neurological deterioration, NIHSS

## Abstract

**Background:**

The incremental value of routine admission measurements for predicting early neurological deterioration after endovascular thrombectomy (EVT) remains unclear.

**Methods:**

This retrospective single-center reanalysis used a 2020–2022 registry. END48 was defined as an increase of at least 4 National Institutes of Health Stroke Scale (NIHSS) points from admission to the fixed 48-h postoperative assessment. The conservative primary cohort excluded an unresolved longitudinal data segment. A revision-stage locked five-predictor logistic regression model included admission NIHSS scores, age, glucose levels, white blood cell count, and ln triglyceride value, with the NIHSS-only model serving as the comparator. Internal validation used 1,000 bootstrap samples and paired nested intervals of 1,000 outer × 100 inner samples.

**Results:**

Of the 253 records representing 251 unique patients, 214 adults entered the primary cohort; 51 (23.8%) had END48. The primary model had an apparent area under the curve (AUC) of 0.651 and an optimism-corrected AUC of 0.601 (0.511 to 0.723). The corrected AUC difference vs. the NIHSS-only model was 0.038 (−0.025 to 0.171). The corrected Brier score was 0.180, compared to a prevalence-only reference of 0.182, resulting in a Brier skill score of 0.9%. The corrected primary calibration slope was 0.731 (0.519 to 0.892). Incremental net benefit was −0.015, 0.036, and 0.013 at 10%, 20%, and 30% thresholds, respectively; all corresponding 95% confidence intervals crossed zero. *Post-hoc* Riley assessments across plausible model-fit assumptions indicated that the available cohort was below the sample size desirable for model development. The reconstructed cohort sensitivity analysis did not change the overall interpretation.

**Conclusion:**

Discrimination was limited, improvement in probability accuracy was marginal, and incremental discrimination and net benefit were uncertain. The model should be regarded as exploratory rather than clinically implementable; independent validation and improved outcome ascertainment are required.

## 1 Introduction

EVT improves functional outcomes in appropriately selected patients with acute ischemic stroke caused by large-vessel occlusion (1). Nevertheless, neurological deterioration remains clinically important. Definitions vary regarding the reference examination, the NIHSS threshold, and assessment window; however, early deterioration in acute ischemic stroke and after thrombectomy is consistently associated with unfavorable outcomes (2, 3). For instance, an EVT nomogram study used an increase of at least 2 NIHSS points within 72 h after stroke onset (4). Its endpoint differs from deterioration present at a fixed 48-h postoperative examination and cannot be considered as directly interchangeable.

The admission NIHSS score is an established measure of initial stroke severity and prognosis (5). Admission hyperglycemia has been associated with poor outcomes after thrombectomy (6). White blood cell (WBC) count evidence from the WAKE-UP trial concerns an intravenous-thrombolysis population, rather than direct validation of a post-EVT predictor (7). Associations involving triglycerides vary across stroke settings: Lower concentrations have been associated with greater initial severity, whereas other studies examine subsequent outcomes across population and metabolic contexts (8, 9). These associations motivate investigation but do not establish causal effects or incremental predictive performance.

We evaluated a revision-stage locked five-predictor model using the NIHSS score, age, glucose level, WBC count, and ln triglyceride value against an NIHSS-only logistic regression model. The aim was to quantify discrimination, probability accuracy, calibration, and decision consequences for END48, following contemporary prediction-model guidance (10). The intended research population comprised adults receiving EVT. Potential users of any future externally validated version would be stroke clinicians; no monitoring or management strategy was evaluated in this study. No model-guided management pathway or clinical impact was tested.

## 2 Methods

### 2.1 Study design and cohort assembly

This retrospective study analyzed data from the EVT registry of the Department of Neurology, Shanxi Provincial People's Hospital, Shanxi Medical University, Taiyuan, China, from 1 January 2020 to 31 December 2022. The research team confirmed that the 253 supplied records represented the complete registered EVT series during this period. Eligible patients were adults aged 18 years with imaging-confirmed acute ischemic stroke who underwent EVT and had an ascertainable admission-to-fixed-48-h NIHSS change.

Mutually exclusive exclusion criteria were applied sequentially: duplicate records, age under 18 years, missing or invalid canonical admission NIHSS scores, missing or invalid fixed 48-h NIHSS scores under documented adjudication, and all remaining patients within original identifiers 205–210. This final step removed the entire unresolved segment from the primary cohort; patient identifier 210 had already been excluded due to unavailable follow-up and was not counted again. Missing laboratory predictors did not determine outcome eligibility. The study flow is shown in Figure 1.

**Figure 1:**
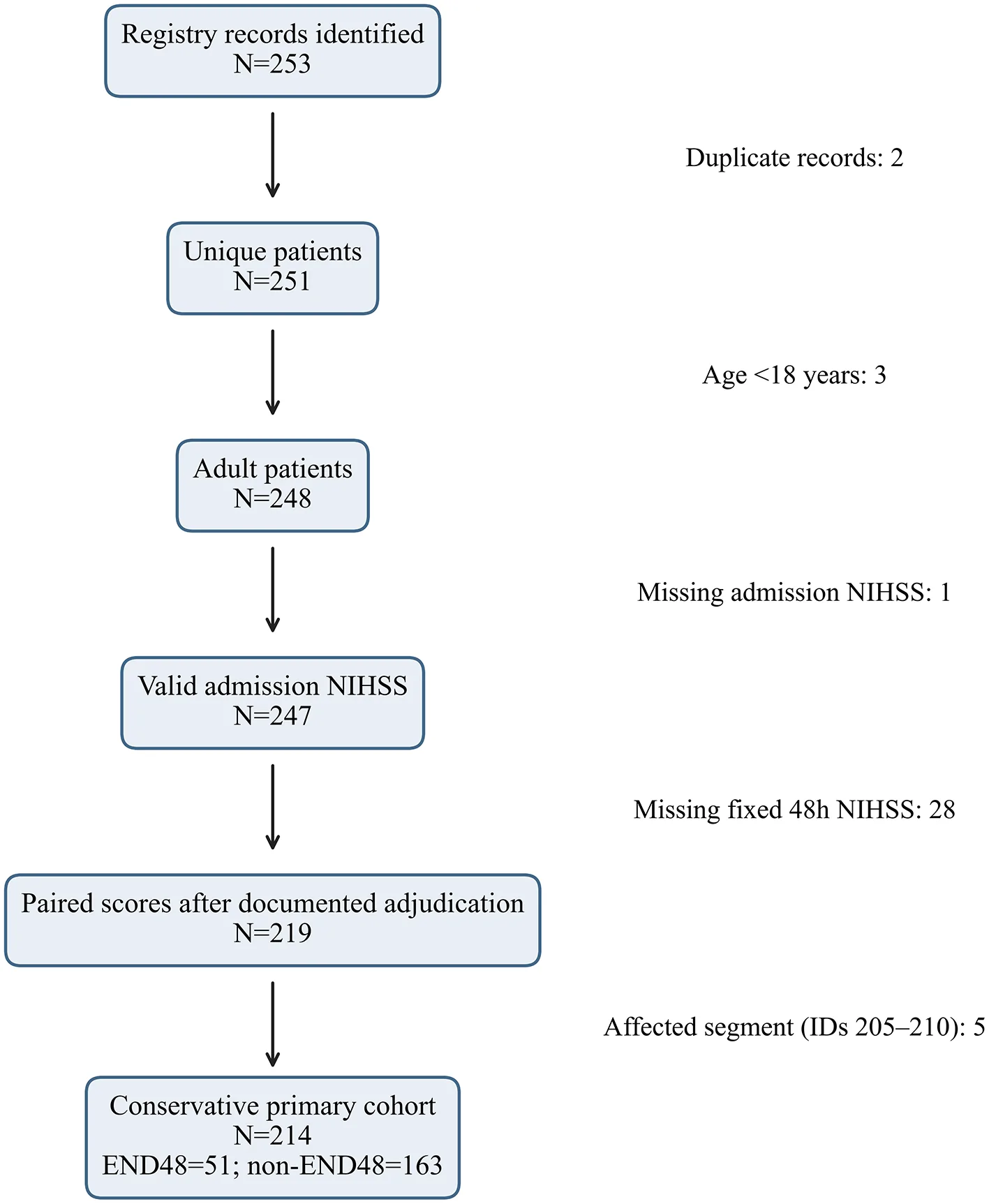
Study flow diagram. Registry records, unique patients, and unique adults are distinguished. The mutually exclusive sequence is 253 – 2 = 251; 251 – 3 = 248; 248 – 1 – 28 = 219; 219 – 5 = 214. The intermediate paired-score cohort includes internally reconstructed records; the primary analysis conservatively excluded the unresolved affected identifier segment. Identifier 210 was already excluded for unavailable follow-up, avoiding double counting. Final END48/non-END48 counts are 51/163.

Routine care and co-treatments were not assigned by this study. Reliable patient-level admission/procedure dates were unavailable for a temporal split, so validation was internal only.

### 2.2 Data audit and revision-stage decisions

Raw data files and all previous audit outputs were retained unchanged. The research team confirmed duplicate record pairs 12/13 and 52/56. The recorded outcome-independent retention rule selected greater completeness of outcome and primary predictors, with the lower identifier breaking ties; records 12 and 52 were retained. Three records with documented ages of 14, 14, and 15 years were retained and subsequently excluded under the adult eligibility.

The canonical admission NIHSS score was extracted from source column J; the fixed 48-h postoperative NIHSS score was obtained from column HM. The research team's source chart verified record 249 as 25 rather than 255. Scores of 35 were retained as valid NIHSS values. In the affected segment, six repeated admission fields in original longitudinal rows 206–210 matched those in rows 205–209: 29 exact numerical matches and one matching missing pair. The earlier internal-consistency reconstruction simultaneously reassigned the original HG: HX blocks 206–210 to 205–209 and cleared the unrecoverable block for 210. The conservative primary analysis excluded the entire affected identifier segment because the longitudinal reassignment, although strongly supported by internal database consistency, was not independently source chart verified for every follow-up measurement. The reconstructed cohort was analyzed only as a sensitivity analysis.

The new frozen dataset applied only the exclusion of identifiers 205–210 to the prior frozen cohort; no other patient values were cleaned again. The SHA256, exclusions, and modeling decisions were recorded. The five-predictor set, ln triglyceride value, decision thresholds, and resampling procedures were retained unchanged. These represent revision-stage decisions, rather than an originally registered prospective protocol. Earlier registry results were already known when the revised model specification was locked. Reporting was mapped to the TRIPOD+AI statement, which also covers multivariable regression models (11).

### 2.3 Outcomes, predictors, and clinical descriptions

END48 was recalculated as fixed postoperative 48-h NIHSS minus canonical admission NIHSS ≥4 points. Both measurements were required to fall within the valid range of 0–42. The NIHSS is a standard neurological deficit scale (12). This endpoint describes deterioration present at the fixed examination; it does not capture transient deterioration occurring during the preceding 48 h. No missing outcome was imputed. Assessor qualifications and blinding were insufficiently documented to permit formal evaluation.

The comparator model used the admission NIHSS scores alone. The primary model added age (years), admission glucose levels (mmol/L), WBC count ( × 10^9^/L), and ln triglyceride value (triglycerides measured in mmol/L). Canonical admission variables were defined during the prespecified data-audit process; duplicated longitudinal fields were not used as additional predictors. Admission NIHSS scores, age, glucose levels, and WBC count entered the model linearly. The earlier revision-stage distribution rule led to natural-log triglycerides; this transformation was retained without reassessment or reselection in the conservative cohort. Fasting status was not established for the canonical admission glucose measurement.

Baseline summaries used observed values before imputation. Table 1 describes admission NIHSS scores, age, glucose levels, WBC count, triglyceride levels, prestroke modified Rankin Scale (mRS) (13) scores, recorded sex, smoking history, and alcohol consumption history. Smoking/alcohol fields describe recorded medical history, rather than current exposure intensity. Because comorbidity documentation had a substantial unknown status, its explicit present/absent/unknown coding and classifiable denominators are reported in Supplementary Table S10. Ambiguous summary codes were not treated as diagnoses, and omission from a disease list was not construed as absence. Detailed imaging/procedure fields with insufficiently established definitions were excluded.

**Table 1:** Baseline characteristics.

Characteristic	All *N* = 214	END48 *N* = 51	Non-END48 *N* = 163
Admission NIHSS (points)	14.00 (11.00–20.00); *n* = 214	18.00 (11.00–21.50); *n* = 51	14.00 (11.00–19.00); *n* = 163
Age (years)	63.00 (53.00–69.00); *n* = 214	60.00 (51.00–67.00); *n* = 51	63.00 (55.50–70.00); *n* = 163
Admission glucose level (mmol/L)	7.05 (6.10–8.70); *n* = 198	8.05 (6.62–8.81); *n* = 46	7.00 (6.00–8.60); *n* = 152
Admission WBC count ( × 10^9^/L)	9.34 (7.32–12.12); *n* = 212	10.34 (7.94–13.44); *n* = 51	9.14 (7.03–11.95); *n* = 161
Triglyceride level (mmol/L)	1.23 (0.87–1.79); *n* = 198	1.22 (0.85–1.92); *n* = 43	1.25 (0.89–1.69); *n* = 155
Prestroke mRS (points)	0.00 (0.00–0.00); *n* = 214	0.00 (0.00–0.00); *n* = 51	0.00 (0.00–0.00); *n* = 163
Recorded male sex	144 (67.3%)	34 (66.7%)	110 (67.5%)
Smoking history	108 (50.5%)	23 (45.1%)	85 (52.1%)
Alcohol consumption history	65 (30.4%)	12 (23.5%)	53 (32.5%)

Continuous values are median (IQR) with observed n. Categorical values are recorded as *n* (%). Summaries precede imputation. Detailed comorbidity documentation, including unknown status and classifiable denominators, is in Supplementary Table S10. No *P*-values or predictor selections are based on this table.

Included and excluded unique adults were compared descriptively in Supplementary Table S8, reporting observed denominators, in-hospital outcomes, and continuous-variable standardized mean differences (SMDs). SMDs describe effect magnitude, not statistical significance; accordingly, no *P*-value was used to establish an absence of selection bias or to select predictors.

### 2.4 Sample size, missing data, and model estimation

This was a fixed retrospective cohort. The 51 events and five predictor parameters correspond to 10.2 events per parameter, excluding the intercept; however, this heuristic ratio alone does not establish sample size adequacy (14). We applied the Riley binary-outcome framework using the pmsampsize 1.1.3 with an observed prevalence of 0.238318, five predictor parameters, a target global shrinkage factor of 0.90, an absolute limit of 0.05 on optimism in Nagelkerke *R*^2^, and an overall-risk precision of ± 0.05 at the 95% confidence level (15, 16). Required N was the maximum among the three criteria.

No defensible external anticipated *R*^2^ was identified for the same endpoint and predictor set. Although pmsampsize supports a C-statistic-based approximation, differently defined END outcomes could not justify borrowing an AUC. We therefore evaluated hypothetical Nagelkerke *R*^2^ values of 0.05, 0.10, 0.15, 0.20, 0.30, and 0.40, corresponding to Cox–Snell *R*^2^ values of approximately 0.033–0.267 at this prevalence. The 0.15 value was an illustrative fallback discussed by Riley, not a validated expectation for this study. The maximum Cox–Snell *R*^2^ was derived from the observed outcome prevalence. The current observed model *R*^2^ was not used as an anticipated performance metric. This was a *post-hoc* application of the Riley framework to a fixed retrospective cohort and was not a prospective sample-size determination. Assumptions and all criteria are detailed in Supplementary Table S9.

Missing values for glucose, WBC, and triglycerides were replaced with the development-sample median, with these medians applied unchanged to the test data; triglyceride levels were imputed before taking logarithms. No outcome information was entered into the imputation. This simple approach does not propagate missing-value uncertainty or establish a missing-at-random mechanism. A complete-case sensitivity analysis was retained. Predictor missingness and univariable descriptions are in Supplementary Tables S1, S2.

Both models were fitted using standard maximum-likelihood logistic regression analyses with an intercept. Development-sample centering and scaling aided numerical fitting; reported regression coefficients were returned to original units. No association between *P*-value, AUC comparison, or new data-driven search changed the five predictors. Wald standard errors and 95% confidence intervals were reported; coefficients and the covariance matrix were independently checked against the statsmodels library.

### 2.5 Internal validation and uncertainty

Both models were evaluated across the same 1,000 patient-level non-parametric bootstrap draws. Imputation, predictor scaling, and model fitting were repeated within each bootstrap development sample. Apparent performance in that sample minus performance in the original cohort estimated optimism; its mean was subtracted from the original apparent performance. For the Brier score, the signed correction generally increases prediction error. No random holdout set was reserved (17).

A separate paired nested bootstrap generated corrected percentile 95% confidence intervals: Each of the 1,000 outer patient resamples repeated model fitting and used 100 paired inner samples for optimism correction (18). Differences were calculated between models within each corrected outer replicate before taking percentiles. These intervals quantify internal resampling uncertainty, not external transportability; 100 inner replicates add Monte Carlo approximation error. The base seed was 20,260,904, with deterministic task-derived seeds and saved draws. Supplementary Table S5 reports computational execution details and numerical exceptions.

### 2.6 Performance and decision analysis

We evaluated AUC, Brier score, Brier skill Score, calibration-in-the-large, and calibration slope (19). Calibration-in-the-large estimated an intercept with the predicted logit as an offset and slope fixed at 1; calibration slope estimated an intercept jointly. These fits diagnosed predictions without updating them. Apparent calibration for fitted unpenalized logistic regression is ideal by construction. A LOWESS apparent calibration curve and pointwise bootstrap optimism corrections were plotted alongside the fitted-risk distribution. Null Brier was p(1 – p), using cohort prevalence p; the Brier skill score was 1–Brier/null Brier. Nested skill intervals used each outer sample's prevalence.

Decision-curve net benefit (NB) was TP/N – (FP/N) × pt/(1 – pt), with predicted risk ≥pt positive (20, 21). The treat-none NB was 0; treat-all NB was p – (1 – p) × pt/(1 – pt). Thresholds of 10%, 20%, and 30% were retained from the earlier framework as clinically considered scenarios, without cutoff optimization; they are not established intervention thresholds. Curves covered 5–40%, highlighting 10–30%. We report corrected NB and paired ΔNB, as well as apparent confusion counts, diagnostic ratios, and consequences per 100. Independently corrected consequences are estimates, not observed counts or one reconstructed confusion matrix. Direct formula checks verified classification counts, NB identities, and hand-calculated examples.

### 2.7 Sensitivity analyses

Sensitivity analyses S1 and S2 separately replaced admission NIHSS score and/or age with restricted cubic splines using four development-sample knots at the 5th, 35th, 65th, and 95th percentiles, giving three degrees of freedom for that predictor and seven predictor parameters overall. Overall and non-linear Wald tests used three and two degrees of freedom. Spline curves with pointwise Wald 95% confidence intervals were generated for descriptive visualization.

Sensitivity analysis S3 used a ridge regression model with the same five predictors. Minimum five-fold validation log loss selected λ from 0.001, 0.01, 0.1, 1, 10, 100, and 1000. Preprocessing occurred within each training fold; duplicate copies of a patient in bootstrap samples stayed in the same fold. S4 used complete cases, and S6 used linear triglyceride levels. Each feasible sensitivity in the conservative cohort used 1,000 bootstrap samples for point correction. No sensitivity analysis replaced the primary model.

Sensitivity analysis S7 used the previously frozen database-internal-consistency reconstructed cohort. Its unchanged, previously QC-passed model results and paired nested intervals were retained rather than recomputed. Comparison with the conservative primary results is descriptive, because the cohorts differ; there is no paired between-cohort confidence interval. Sensitivity analysis S7 tests model robustness to the adjudication assumption, not the correctness of reconstructed measurements. Model specifications and the results are provided in Supplementary Tables S3, S6, S7 as well as Supplementary Figures S1, S2.

Sensitivity analysis S5, an END48-or-death-within-48-h composite, was unavailable because reliable timing of death relative to EVT could not be reconstructed for all relevant patients. In-hospital death was not substituted for 48-h death. Temporal validation and external validation were unavailable, and no *post hoc* probability updating was performed.

## 3 Results

### 3.1 Cohort, clinical characteristics, and sample size

Of the 253 registry records, two duplicates were removed, leaving 251 unique patients. Three minors were excluded, leaving 248 unique adults. One adult lacked a valid admission NIHSS score, and 28 further adults lacked an ascertainable fixed 48-h score under the documented adjudication. The resulting 219-person reconstructed cohort contained five patients from the affected identifier segment; all five were non-events. Their conservative exclusion yielded 214 patients, comprising 51 END48 events (23.8%) and 163 non-events (Figure 1). No primary record belonged to identifiers 205–210.

Table 1 presents observed clinical characteristics. Glucose and triglyceride levels were each missing in 16 patients and WBC count in two; admission NIHSS score and age were complete. Expanded comorbidity summaries retained a substantial unknown status. The 34 excluded unique adults were older in median age (67 vs. 63 years) and had a slightly lower median admission NIHSS score (13 vs. 14). Age and NIHSS SMD magnitudes were 0.367 and 0.239, respectively. Recorded in-hospital outcomes in excluded adults were improvement in 24, death in eight, and self-discharge in two; the corresponding included counts were 168, 43, and three. These outcomes had no reliably established 48-h timing (Supplementary Table S8).

Under the illustrative Nagelkerke R^2^ of 0.15 (Cox–Snell R^2^ approximately 0.100), the required sample size was 425, corresponding to 101.3 expected events. Required sample sizes ranged from 279 to 1,326 across the specified assumptions, all above the available 214. Under the illustrative assumption, the shrinkage, model-fit optimism, and risk-precision criteria required 425, 140, and 279 patients, respectively. The available cohort was smaller than the sample size desirable under the Riley framework, reinforcing the exploratory nature of model development (Supplementary Table S9).

### 3.2 Model estimates and discrimination

Table 2 presents the full five-predictor model estimates. All predictors remained in the model regardless of the *P*-value. The estimated age coefficient was negative; the NIHSS, glucose, WBC, and ln triglyceride coefficients were positive. These estimates represent conditional associations with the defined change-score endpoint, not causal effects.

**Table 2:** Revision-stage locked primary logistic regression model.

Predictor	β	SE	OR (95% CI)	*P*
Intercept	−2.097	1.129	—	0.063
Admission NIHSS score	0.042	0.033	1.043 (0.978–1.112)	0.201
Age	−0.019	0.015	0.981 (0.953–1.010)	0.196
Admission glucose level	0.092	0.059	1.096 (0.976–1.232)	0.122
Admission WBC count	0.065	0.039	1.068 (0.988–1.154)	0.097
ln triglyceride value	0.118	0.328	1.125 (0.592–2.140)	0.719

*N* = 214; events = 51. Original measurement units: ln triglycerides OR is per e-fold increase in triglycerides. Standard maximum likelihood with all five predictors retained. Wald intervals are conditional on median imputation. Full-precision coefficients and covariance are supplied in Supplementary Table S4 and the saved model.

The primary model had an apparent AUC of 0.651 and an optimism-corrected AUC of 0.601 (0.511 to 0.723). The corrected AUC difference vs. the NIHSS-only model was 0.038 (−0.025 to 0.171). The NIHSS-only corrected AUC was 0.563 (0.462 to 0.673). Table 3 reports apparent and corrected performance; Figure 2A displays only apparent ROC curves and apparent AUC. The paired ΔAUC interval includes zero, so added discrimination is unclear.

**Table 3:** Predictive performance.

Metric	Model	Apparent	Optimism corrected (95% CI)
AUC	NIHSS-only	0.575	0.563 (0.462 to 0.673)
AUC	Primary	0.651	0.601 (0.511 to 0.723)
Brier	NIHSS-only	0.179	0.183 (0.151 to 0.211)
Brier	Primary	0.168	0.180 (0.143 to 0.205)
Brier Skill Score (%)	NIHSS-only	1.5	−0.6 (−3.0 to 5.8)
Brier Skill Score (%)	Primary	7.3	0.9 (−3.5 to 13.3)
Calibration in the large	NIHSS-only	0.000	0.013 (−0.025 to 0.039)
Calibration in the large	Primary	0.000	0.018 (−0.022 to 0.046)
Calibration slope	NIHSS-only	1.000	1.125 (−2.444 to 5.547)
Calibration slope	Primary	1.000	0.731 (0.519 to 0.892)
Paired corrected differences			
Primary minus NIHSS-only		Difference (95% CI)	
AUC		0.038 (−0.025 to 0.171)	
Brier		−0.0029 (−0.0227 to 0.0041)	

Both models *N* = 214, events = 51; null Brier = 0.182. Brier Skill Score is expressed as a percentage. Apparent calibration is ideal by construction. Corrected intervals use paired nested percentile bootstrap (1,000 outer × 100 inner). Comparator slope is numerically unstable and must not be interpreted as adequate calibration.

Positive ΔAUC and negative ΔBrier favor primary; both intervals include zero. ΔBrier uses four decimals to retain its small magnitude.

**Figure 2:**
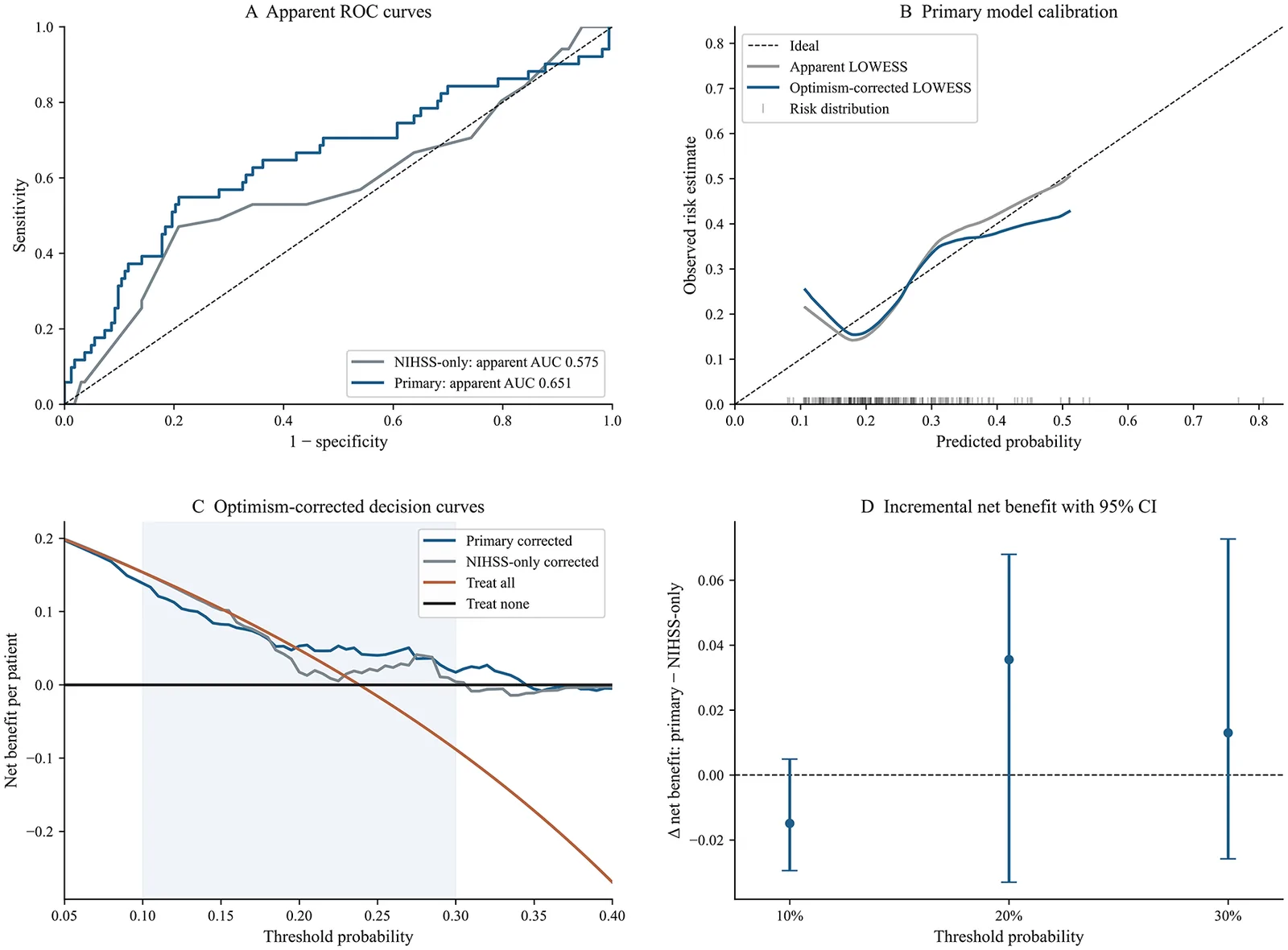
Model performance. **(A)** Apparent ROC curves and apparent AUC only. Optimism-corrected AUC estimates are reported in Table 3. **(B)** Primary apparent LOWESS and pointwise optimism-corrected calibration over the 2.5th−97.5th percentiles of fitted risk; the corrected curve is descriptive without confidence bands. **(C)** Corrected decision curves on the raw NB scale, with treat-all, treat-none, and the highlighted 10–30% interval. **(D)** Corrected primary-minus-NIHSS-only NB differences with paired nested 95% intervals at retained thresholds. *N* = 214; events = 51. Predictions were not *post hoc* recalibrated.

### 3.3 Probability accuracy and calibration

The corrected Brier score was 0.180, compared with a prevalence-only reference of 0.182, giving a Brier skill score of 0.9%. The NIHSS-only corrected Brier score was 0.183. The paired corrected ΔBrier (primary minus comparator) was −0.0029 (−0.0227 to 0.0041); its interval included zero. The primary Brier skill score had a 95% confidence interval of −3.5% to 13.3%.

The corrected primary calibration slope was 0.731 (0.519 to 0.892). The corrected calibration-in-the-large was 0.018 (−0.022 to 0.046). The slope below 1 is consistent with overly extreme fitted risks. The NIHSS-only corrected calibration slope was 1.125 (−2.444 to 5.547), with very unstable resampled test slopes; this is not evidence of adequate comparator calibration. Four comparator inner calibration fits were non-estimable among 100,000 resamples, while all primary and non-calibration metrics were finite. Figure 2B gives descriptive corrected calibration; Table 3 and Supplementary Table S5 retain the numerical limitations.

### 3.4 Threshold performance

Across the retained 10%, 20%, and 30% thresholds, apparent operating characteristics showed the expected trade-off between detecting END48 and reducing positive classifications (Table 4A). At 20%, the primary model had an apparent sensitivity of 0.706 and a specificity of 0.485. Component-wise optimism correction estimated 56.19 positive classifications, 15.47 END48 cases detected, and 8.44 END48 cases missed per 100 patients (Table 4B).

**Table 4A:** Development-sample operating characteristics.

Model	Threshold (%)	Sensitivity	Specificity	PPV	NPV	LR+	LR–
NIHSS-only	10	1.000	0.000	0.238	NE	1.000	NE
NIHSS-only	20	0.706	0.258	0.229	0.737	0.951	1.141
NIHSS-only	30	0.255	0.859	0.361	0.787	1.806	0.868
Primary	10	0.961	0.006	0.232	0.333	0.967	6.392
Primary	20	0.706	0.485	0.300	0.840	1.370	0.607
Primary	30	0.392	0.828	0.417	0.813	2.283	0.734

All values are apparent development-sample estimates at predicted risk ≥ threshold; they are not external-validation estimates. NE indicates an undefined denominator.

**Table 4B:** Optimism-corrected decision consequences and net benefit.

Model	Threshold (%)	Positive classifications/100	END48 detected/100	END48 missed/100	Corrected NB	Incremental NB vs. NIHSS-only (95% CI)
NIHSS-only	10	99.95	23.81	0.10	0.153	Reference
NIHSS-only	20	73.28	16.04	7.87	0.017	Reference
NIHSS-only	30	16.64	5.27	18.63	0.004	Reference
Primary	10	98.36	22.30	1.60	0.139	−0.015 (−0.029 to 0.005)
Primary	20	56.19	15.47	8.44	0.053	0.036 (−0.033 to 0.068)
Primary	30	22.32	7.89	16.02	0.017	0.013 (−0.026 to 0.073)

Each consequence component was corrected independently, and the values do not constitute one reconstructed confusion matrix. NB is on the raw scale. Every paired ΔNB 95% CI crosses zero. No intervention benefit was measured. Full threshold details are in Supplementary Table S11.

The corrected incremental net benefit vs. the NIHSS-only model was −0.015 (95% CI −0.029 to 0.005), 0.036 (−0.033 to 0.068), and 0.013 (−0.026 to 0.073) at 10%, 20%, and 30%, respectively; every interval crossed zero (Table 4B; Figures 2C, D). These estimates do not establish clinical benefit from a model-guided intervention. Full apparent counts, predictive values, likelihood ratios, apparent and corrected consequences, and net benefit intervals are reported in Supplementary Table S11.

### 3.5 Sensitivity analyses

Corrected AUCs were 0.615 for the S1 NIHSS spline model, 0.592 for the S2 age spline model, 0.601 for the S3 ridge regression model, and 0.611 for the S6 linear triglycerides model. S4 included 182 complete cases with 39 events and had a corrected AUC of 0.639 and a Brier score of 0.162. Spline forms and complete-case results are detailed in Supplementary Tables S3, S6 as well as Supplementary Figures S1, S2.

Sensitivity analysis S7 comprised 219 patients with 51 events. Its reconstructed-cohort primary model had a corrected AUC of 0.606, a Brier score of 0.176, a calibration slope of 0.742, and a ΔAUC of 0.043; all three ΔNB intervals crossed zero (Supplementary Table S7). These findings leave the overall interpretation materially unchanged and support model robustness to the adjudication assumption; however, they do not verify the empirical accuracy of the reconstruction. Sensitivity analysis S5 and temporal validation remained unavailable.

## 4 Discussion

### 4.1 Principal findings

In the conservative 214-patient cohort, the revision-stage locked five-predictor model had a corrected AUC of 0.601, with uncertain incremental discrimination over admission NIHSS score alone [ΔAUC 0.038 (−0.025 to 0.171)]. The corrected Brier score of 0.180 was only marginally below the prevalence-only reference of 0.182; the Brier skill score was 0.9%. All threshold-specific 95% confidence intervals for ΔNB crossed zero. These findings do not establish clinically useful incremental predictive value.

Excluding the entire unresolved segment ensured that the primary analysis remained independent of the longitudinal data reconstruction. The reconstructed-cohort sensitivity analysis yielded similar conclusions, but internal database matching is not a substitute for independent source chart verification. END definitions also differ across previous studies (2–4); differences in endpoint timing, severity, and case mix limit direct comparisons of AUCs.

### 4.2 Sample size, optimism, and calibration

The *post hoc* Riley assessment required 425 patients under the illustrative Nagelkerke *R*^2^ of 0.15, and even the overall-risk precision criterion required 279 patients. All evaluated assumptions exceeded the size of the available cohort. These conditional calculations support concerns about precision and model overfitting; however, they neither establish a true anticipated *R*^2^ nor retrospectively provide a prospective sample-size justification (15, 16). Reducing the primary model to five predictor parameters did not remove the limitations of a small fixed event count (14).

The corrected calibration slope of 0.731 indicated model overfitting despite its modest complexity. The apparent-to-corrected AUC reduction and broad paired intervals reinforce predictive uncertainty. Comparator slope estimates were unstable when fitted risks varied little, and the ridge regression sensitivity analysis also had an unstable corrected slope. Neither model supports the estimation of reliable individual risk probabilities. Calibration assessments should accompany evaluations of discrimination and be reassessed in independent external populations (19).

### 4.3 Decision consequences

Threshold analyses express the trade-off between missed deterioration and false-positive classifications under specified relative harms. They do not identify an optimal threshold or demonstrate clinical benefit from increasing monitoring, imaging, or treatment (20, 21). The 20% scenario had a positive point estimate for incremental NB, but its 95% confidence interval crossed zero, as did those at 10% and 30%. Prospective evaluation of a defined management response would be necessary following external validation; bedside implementation is not justified by these internal exploratory results.

### 4.4 Clinical associations and the change-score endpoint

The admission NIHSS score coefficient requires particular care because the admission NIHSS score is both a predictor and the baseline value subtracted in the outcome definition. Mathematical coupling, measurement error, and regression to the mean may influence its observed association. This does not automatically invalidate NIHSS as a predictor, but it limits any mechanistic interpretation of its coefficient sign or spline curve shape. Likewise, the negative coefficient estimate for age should not be read as a protective causal effect.

Glucose, inflammatory measures, and triglycerides have been associated with stroke severity or clinical outcomes in prior studies (6–9). Our results do not show that measuring or therapeutically modifying these factors improves END48 prediction or prevents clinical deterioration. The WBC evidence is partly from a different treatment setting, and triglyceride findings do not establish a treatment target. Spline analyses are diagnostic checks of functional form, rather than data-driven searches for biological thresholds.

### 4.5 Limitations

This single-center, retrospective cohort was limited by a limited number of events, incomplete laboratory values, and the availability of internal validation only. Median imputation ignores missing-value uncertainty; complete cases change the case mix and event prevalence and cannot establish that the missingness mechanism is ignorable. Comorbidity coding inconsistencies required conservative text-based descriptions with explicit recording of unknown status, limiting the interpretation of apparent prevalence differences. Detailed imaging, reperfusion, and procedural covariates were not sufficiently standardized for additional descriptive analysis.

The 34 excluded adults were older on average and median, and they had more missing admission glucose levels than included patients. Their recorded hospital deaths (eight of 34) cannot be classified reliably as deaths within 48 h. Exclusion of missing fixed assessments can therefore produce survivor and outcome-ascertainment bias, regardless of the size of observed baseline differences. Excluding identifiers 205–210 lowers reliance on inferred reconstruction but does not solve missing-outcome bias. Sensitivity analysis S7 evaluates only robustness to the adjudication assumption.

Because the revision-stage predictor set was specified after the results from earlier analyses of the same registry were already known, residual model-selection optimism cannot be excluded despite locking the revised model specification before rerunning the predictive analyses. Bootstrap validation repeats the locked fitting pipeline, not the earlier, incompletely reconstructable model-selection history. It therefore cannot be assumed to remove all selection optimism. The endpoint also misses transient deterioration that resolves before the fixed examination. Assessor blinding, measurement consistency, and original protocol/registration information were not sufficiently documented. Reliable temporal validation, external validation, demographic subgroup performance, and formal equity assessment were unavailable. Finally, no model-guided clinical impact or causal treatment effect was evaluated.

## 5 Conclusion

In this conservative analysis of 214 adults with 51 END48 events, limited discrimination and marginal probability-accuracy improvement were observed with a five-predictor model. Incremental discrimination and net benefit beyond the admission NIHSS score alone were uncertain. The *post hoc* Riley sample size assessment and the observed calibration results reinforce the exploratory nature of model development. The model requires independent validation and improved outcome ascertainment before any consideration of clinical implementation.

## Data Availability

The dataset contains de-identified clinical data from patients with acute ischemic stroke treated at Shanxi Provincial People's Hospital. Due to institutional policy and patient privacy protection requirements, the dataset is not publicly available. Access to the data is restricted and may be granted by the corresponding author upon reasonable request, subject to approval by the Ethics Committee of Shanxi Provincial People's Hospital and compliance with applicable data protection regulations. The data may only be used for scientific research purposes and cannot be shared with third parties without permission. Requests to access the datasets should be directed to the corresponding author, Yaxuan Sun (yaxuansjjr@163.com).
