# Supplementary material for: Development and internal validation of a parsimonious model for neurological deterioration at 48 h after endovascular thrombectomy
Source: Front Neurol. 2026 Oct 16;17:1888204. doi: 10.3389/fneur.2026.1888204 (PMC13719573; doi:10.3389/fneur.2026.1888204)
Supplement: Supplementary file 1 [file Table_1.docx]

Supplementary Methods and Results

Conservative primary: N=214, END48=51. Original frozen values are retained except that all identifiers 205–210 are excluded. CSV SHA256: 6d5874c4d53446bb749c845884591251addd453f01f89f2ca23ac447e128ac01. Full-precision CSV/NPZ outputs and the workbooks accompany the manuscript. No source identifiers or patient names are included in these manuscript tables.

# S1 Missingness and preprocessing

Supplementary Table S1 Predictor missingness

| **Predictor** | **N** | **Missing** | **Missing %** |
| --- | --- | --- | --- |
| Admission NIHSS | 214 | 0 | 0.0 |
| Age | 214 | 0 | 0.0 |
| Admission glucose | 214 | 16 | 7.5 |
| Admission WBC | 214 | 2 | 0.9 |
| Triglycerides | 214 | 16 | 7.5 |

No outcome was imputed. Raw-scale full-sample medians in the order NIHSS, age, glucose, WBC, triglycerides were 14.000, 63.000, 7.050, 9.345, 1.235. Each resample computes its own development medians before applying ln triglycerides, then its own centring and scaling. Test data use those stored values unchanged. ln triglycerides was inherited from the locked earlier reanalysis, not newly chosen in the conservative cohort. All observed triglycerides were positive; nonpositive input is outside the logarithmic preprocessing rule.

# S2 Univariable associations

Supplementary Table S2 Exploratory univariable associations

| **Predictor** | **N** | **Events** | **OR (95% CI)** | **P** |
| --- | --- | --- | --- | --- |
| Admission NIHSS | 214 | 51 | 1.054 (0.992 to 1.120) | 0.087 |
| Age | 214 | 51 | 0.978 (0.953 to 1.005) | 0.104 |
| Admission glucose | 198 | 46 | 1.118 (1.001 to 1.248) | 0.048 |
| Admission WBC | 212 | 51 | 1.093 (1.017 to 1.175) | 0.016 |
| ln(TG) | 198 | 43 | 1.517 (0.835 to 2.755) | 0.172 |

Observed complete cases for each predictor; no imputation. These associations were not used for predictor selection and are not causal effects.

# S3 Sensitivity analyses

S1 and S2 separately use NIHSS or age restricted cubic splines with four development-sample knots at 5%, 35%, 65%, 95%. In addition to x, the two nonlinear basis terms are d_j(x)={(x−k_j)₊³−[(k4−k_j)/(k4−k3)](x−k3)₊³+[(k3−k_j)/(k4−k3)](x−k4)₊³}/(k4−k1)², j=1,2. This yields linear tails and three degrees of freedom for the spline predictor. Other predictors retain their primary functional forms.

S1_NIHSS_RCS: full-sample knots 6.00, 12.00, 18.00, 22.00; overall Wald P=0.169; nonlinear Wald P=0.208. Knots and preprocessing are re-estimated in development resamples. These P values do not select the primary model.

S2_Age_RCS: full-sample knots 42.65, 57.00, 67.00, 80.35; overall Wald P=0.335; nonlinear Wald P=0.410. Knots and preprocessing are re-estimated in development resamples. These P values do not select the primary model.

S3 ridge minimises five-fold validation log loss, grouping copies of the same patient within folds and estimating imputation/scaling in each tuning training fold. λ grid: 0.001, 0.01, 0.1, 1, 10, 100, 1000; final full-sample λ=100. The intercept is unpenalised. S4 is complete case, S6 linear triglycerides, and S7 the prior reconstructed cohort. S5 is unavailable: recorded hospital death cannot substitute for reliably timed death within 48 hours. No temporal validation was performed.

Supplementary Table S3 Corrected sensitivity performance

| **Specification** | **N** | **Events** | **AUC** | **Brier** | **CITL** | **Slope** |
| --- | --- | --- | --- | --- | --- | --- |
| S1 NIHSS RCS | 214 | 51 | 0.615 | 0.181 | −0.004 | 0.684 |
| S2 age RCS | 214 | 51 | 0.592 | 0.182 | 0.021 | 0.658 |
| S3 ridge | 214 | 51 | 0.601 | 0.181 | 0.012 | 2.684 |
| S4 complete case | 182 | 39 | 0.639 | 0.162 | 0.006 | 0.770 |
| S6 linear TG | 214 | 51 | 0.611 | 0.175 | 0.015 | 0.795 |
| S7 NIHSS-only | 219 | 51 | 0.563 | 0.179 | 0.008 | 2.757 |
| S7 five-predictor | 219 | 51 | 0.606 | 0.176 | 0.015 | 0.742 |

All corrected point estimates use 1,000 resamples. S7 retains unchanged prior QC-passed results. No primary replacement or between-cohort paired inference was made. Corrected ridge and S7 comparator slopes are unstable, not evidence of reliable calibration. NB results at full precision are in the supplement workbook.

# S4 Full model equation and coefficients

η = −2.096526732059 + 0.041869340347 × admission NIHSS − 0.019019654536 × age + 0.091856793197 × admission glucose + 0.065492311306 × admission WBC + 0.118054450063 × ln(triglycerides). Predicted probability P=1/[1+exp(−η)]. Units are NIHSS points, age years, glucose mmol/L, WBC ×10⁹/L and triglycerides mmol/L. Impute on the raw scale with the S1 medians before logarithmic transformation. The ln triglycerides OR is per e-fold increase; for doubling, OR=exp(β_lnTG×ln 2).

Supplementary Table S4 Original-unit primary coefficients

| **Term** | **β** | **SE** |
| --- | --- | --- |
| Intercept | −2.096526732059 | 1.129033090 |
| Admission NIHSS | 0.041869340347 | 0.032735687 |
| Age | −0.019019654536 | 0.014722729 |
| Admission glucose | 0.091856793197 | 0.059412878 |
| Admission WBC | 0.065492311306 | 0.039497318 |
| ln triglycerides | 0.118054450063 | 0.327856688 |

Wald ORs and confidence intervals are in main Table 2 and the full-precision coefficient CSV. Median imputation uncertainty is not included in these Wald standard errors.

NIHSS-only: η=−1.976516014295 + 0.052872351034×admission NIHSS. Equations are supplied for reproducibility, not clinical deployment. Full covariance, medians and scaling are retained in 03_analysis/conservative_v2/model_1_parameters.npz; original-unit covariance is also in Table_S4_covariance.csv.

# S5 Bootstrap validation and numerical stability

For any metric M, corrected performance is M(D,fitted D)−mean_b{M(Db,fitted Db)−M(D,fitted Db)}. AUC uses half credit for ties; Brier is mean squared probability error. Shared patient indices pair the two models. Nested intervals resample outer datasets, refit models and subtract their own 100-inner mean optimism before taking 2.5th and 97.5th percentiles of 1,000 corrected outer estimates. Differences are formed within outer draws. This is not resampling fixed predictions or treating optimism alone as an independent test sample. Outer prevalence defines the null Brier for each skill estimate.

Calibration-in-the-large uses an offset with slope fixed at 1; slope is estimated jointly with an intercept. No diagnostic calibration fit changes predictions. LOWESS uses fraction 0.75, no robust iterations and an original fitted-risk grid restricted to 2.5th–97.5th percentiles. Mean development-minus-test curve differences are subtracted pointwise. Out-of-range interpolation is missing, and finite pointwise counts are saved. The corrected smooth curve is descriptive without confidence bands.

All 1,000 main replicates and 1,000 outer estimates were finite. Four comparator inner calibration fits among 100,000 were non-estimable; both calibration metrics were missing in these joint diagnostic failures. The four affected outer inner means used 99 values; all other metric means used 100. All primary calibration and all AUC/Brier/NB values were finite. Comparator main test slopes ranged from −381.612 to 308.564; 38 had absolute value >10. No finite extremes were trimmed. The corrected comparator slope 1.125 is therefore fragile.

Component-wise correction can cross logical bounds: the NIHSS-only corrected specificity at 10% was −0.000321512. It is retained in the workbook, not clipped. The main table uses coherent apparent counts/ratios and separately labels corrected consequences and NB. Undefined ratios are NE. Main threshold comparison intervals are paired; sensitivity point estimates have no newly calculated nested intervals, except the retained S7 intervals from the earlier run.

Supplementary Table S5 Validation execution

| **Component** | **Execution** |
| --- | --- |
| Main point correction | 1,000 shared patient resamples |
| Corrected uncertainty | 1,000 paired outer × 100 paired inner samples |
| Calibration exceptions | Four comparator inner diagnostic failures; affected means use 99 |
| Conservative sensitivities | S1/S2/S3/S4/S6 each 1,000 bootstrap samples |
| S7 reconstructed cohort | Retained QC-passed 1,000 point and 1,000×100 nested results |
| Seed | 20260904; deterministic task-derived seeds |
| Independent verification | statsmodels coefficients/covariance; sklearn AUC; direct Brier; confusion counts; DCA identities |

# S6 Complete-case analysis

Supplementary Table S6 Complete-case coefficients

| **Term** | **Primary β** | **Complete-case β** |
| --- | --- | --- |
| Intercept | −2.096527 | −2.162422 |
| admission NIHSS | 0.041869 | 0.009598 |
| age | −0.019020 | −0.024487 |
| admission glucose | 0.091857 | 0.141704 |
| admission WBC | 0.065492 | 0.097646 |
| ln(triglycerides) | 0.118054 | 0.063845 |

Primary N=214, events=51; complete-case N=182, events=39. Corrected AUC=0.639; Brier=0.162; CITL=0.006; slope=0.770. Comparisons reflect changed case mix and prevalence as well as missingness handling.

# S7 Database-internal-consistency reconstructed cohort

The conservative primary analysis excluded the entire affected identifier segment because the longitudinal reassignment, although strongly supported by internal database consistency, was not independently source-chart verified for every follow-up measurement. The reconstructed cohort was analysed only as a sensitivity analysis. The 29 exact numeric matches and one matching missing pair supported the earlier deterministic simultaneous HG:HX reassignment, but did not independently authenticate every postoperative value. Of identifiers 205–210, five entered the previous 219-person frozen cohort and none had END48; identifier 210 was already excluded for missing follow-up. The reconstructed frozen CSV SHA256 is 77b43291229886195d494b48af2ad7daf330518404215b46ea7c8ee7eb1c49ed.

Supplementary Table S7 Conservative versus reconstructed cohorts

| **Cohort** | **N/events** | **AUC** | **Brier** | **Slope** | **ΔAUC (95% CI)** |
| --- | --- | --- | --- | --- | --- |
| Conservative primary | 214/51 | 0.601 | 0.180 | 0.731 | 0.038 (−0.025 to 0.171) |
| S7 reconstructed | 219/51 | 0.606 | 0.176 | 0.742 | 0.043 (−0.021 to 0.181) |

Metrics are optimism corrected. ΔAUC compares the five-predictor model with NIHSS-only within each cohort. These are not confidence intervals for between-cohort differences.

Supplementary Table S7 continued Paired incremental net benefit

| **Cohort** | **Threshold** | **ΔNB (95% CI)** |
| --- | --- | --- |
| Conservative primary | 10% | −0.015 (−0.029 to 0.005) |
| Conservative primary | 20% | 0.036 (−0.033 to 0.068) |
| Conservative primary | 30% | 0.013 (−0.026 to 0.073) |
| S7 reconstructed | 10% | −0.019 (−0.028 to 0.009) |
| S7 reconstructed | 20% | 0.043 (−0.030 to 0.068) |
| S7 reconstructed | 30% | 0.023 (−0.026 to 0.071) |

The prior QC-passed S7 outputs were reused unchanged, not newly refitted. All intervals cross zero, leaving overall interpretation materially unchanged. This tests robustness to the adjudication assumption, not reconstruction validity.

# S8 Included versus excluded unique adults

The comparison starts with 248 unique adults after duplicate/minor exclusions: 214 included and 34 excluded. Excluded adults comprise one unavailable baseline, 28 unavailable fixed follow-ups under documented adjudication and five further segment exclusions. Duplicate records and minors are not included in either adult comparison denominator. Only unchanged canonical admission/history/hospital-outcome fields were used; reconstructed follow-up values did not enter these descriptors.

Supplementary Table S8 Included versus excluded adults

| **Characteristic** | **Included N=214** | **Excluded N=34** |
| --- | --- | --- |
| Admission NIHSS (points) | 14.00 (11.00–20.00); n=214 | 13.00 (11.00–18.00); n=33 |
| Age (years) | 63.00 (53.00–69.00); n=214 | 67.00 (56.00–75.00); n=34 |
| Admission glucose (mmol/L) | 7.05 (6.10–8.70); n=198 | 6.80 (5.65–8.44); n=20 |
| Admission WBC (×10⁹/L) | 9.34 (7.32–12.12); n=212 | 9.62 (6.91–12.07); n=34 |
| Triglycerides (mmol/L) | 1.23 (0.87–1.79); n=198 | 1.11 (0.84–1.42); n=28 |
| Prestroke mRS (points) | 0.00 (0.00–0.00); n=214 | 0.00 (0.00–0.00); n=33 |
| Recorded male sex | 144/214 (67.3%); u=0 | 21/34 (61.8%); u=0 |
| Hypertension, documented | 123/150 (82.0%); u=64 | 23/29 (79.3%); u=5 |
| Diabetes, documented | 64/106 (60.4%); u=108 | 12/19 (63.2%); u=15 |
| Atrial fibrillation, documented | 57/95 (60.0%); u=119 | 11/16 (68.8%); u=18 |
| Coronary artery disease, documented | 32/76 (42.1%); u=138 | 5/12 (41.7%); u=22 |
| Smoking history | 108/214 (50.5%); u=0 | 16/33 (48.5%); u=1 |
| Alcohol history | 65/214 (30.4%); u=0 | 6/33 (18.2%); u=1 |
| Hospital outcome: improved | 168/214 (78.5%) | 24/34 (70.6%) |
| Hospital outcome: death | 43/214 (20.1%) | 8/34 (23.5%) |
| Hospital outcome: self-discharge | 3/214 (1.4%) | 2/34 (5.9%) |

Continuous values: median (IQR); n observed. Categorical values: positive/observed n (%); u=unknown n. Comorbidities use explicit documentation or explicit absence; omission from other disease lists is unknown. Percentages among classifiable histories are not fully ascertained disease prevalence. Sex, smoking and alcohol are recorded categories/history. Summaries precede imputation. No P values or predictor selection are based on this table. Hospital outcomes are recorded by discharge; death timing relative to EVT is unknown.

Supplementary Table S8 continued Continuous-variable SMDs

| **Variable** | **Absolute SMD** |
| --- | --- |
| Admission NIHSS | 0.239 |
| Age | 0.367 |
| Admission glucose | 0.212 |
| Admission WBC | 0.160 |
| Triglycerides | 0.190 |
| Prestroke mRS | 0.065 |

Absolute difference in observed means divided by the square root of the average of the two observed variances. SMDs are descriptive, not tests of significance or absence of selection bias. For skewed or ordinal variables, interpret alongside the medians/IQRs.

# S9 Post-hoc Riley sample-size assessment

Actual R pmsampsize 1.1.3 calculations used prevalence 0.238317757, five predictor parameters, shrinkage 0.90, maximum optimism of 0.05 in Nagelkerke R² and 95% overall-risk precision ±0.05. Maximum Cox–Snell R² at this prevalence is 0.666551265. No suitable external anticipated R² was identified; the hypothetical grid below is a sensitivity analysis, not observed performance. Riley’s no-information illustrative Nagelkerke value of 0.15 is conditional, not validated for END48. Although a C-statistic option exists, evidence from differently defined END outcomes was not borrowed. See main references 15 and 16.

Supplementary Table S9 Riley criteria across assumed performance

| **Nag R²** | **CS R²** | **N shrink** | **N fit** | **N risk** | **Required N** | **Expected events** |
| --- | --- | --- | --- | --- | --- | --- |
| 0.05 | 0.033328 | 1326 | 145 | 279 | 1326 | 316.0 |
| 0.10 | 0.066655 | 650 | 143 | 279 | 650 | 154.9 |
| 0.15 | 0.099983 | 425 | 140 | 279 | 425 | 101.3 |
| 0.20 | 0.133310 | 312 | 138 | 279 | 312 | 74.4 |
| 0.30 | 0.199965 | 199 | 132 | 279 | 279 | 66.5 |
| 0.40 | 0.266621 | 143 | 127 | 279 | 279 | 66.5 |

Nag=Nagelkerke; CS=Cox–Snell. Expected events=required N×51/214, not a separate fixed event-count guarantee. The accompanying Riley workbook reports expected and upward-rounded event counts for every criterion, including criterion-specific shrinkage. All required N exceed the available 214.

At the illustrative Nagelkerke R² of 0.15 (Cox–Snell R² approximately 0.100), the required sample size was 425, corresponding to 101.3 expected events. Required sample sizes ranged from 279 to 1326 over the specified assumptions, all above the available 214. The sample size for each scenario is the largest of the shrinkage, fit-optimism and overall-risk precision criteria. The actual package outputs were independently checked with the published algebra. This was a post-hoc application to a fixed retrospective cohort, not a prospective determination or a claim that the study was adequately powered. Current observed model R² was not substituted for anticipated performance.

# S10 Primary-cohort comorbidity documentation

Comorbidities were not model predictors. A named condition in a clearly labelled field was coded present; explicit absence without a conflicting positive entry was coded absent. Blank fields, omission from other disease lists and conflicting records were unknown. Percentages therefore use only classifiable records and must not be interpreted as fully ascertained prevalence.

Supplementary Table S10 Primary-cohort comorbidity documentation

| **Characteristic** | **Present n** | **Absent n** | **Unknown n** | **Classifiable denominator** | **Present among classifiable %** |
| --- | --- | --- | --- | --- | --- |
| Hypertension | 123 | 27 | 64 | 150 | 82.0 |
| Diabetes mellitus | 64 | 42 | 108 | 106 | 60.4 |
| Atrial fibrillation | 57 | 38 | 119 | 95 | 60.0 |
| Coronary artery disease | 32 | 44 | 138 | 76 | 42.1 |

Primary cohort N=214. Present + absent equals the classifiable denominator; classifiable + unknown equals 214 for each condition.

# S11 Detailed threshold analysis

The six parts below retain every threshold detail removed from the condensed main Table 4. Apparent estimates describe this development sample. Corrected quantities use the saved 1,000-sample bootstrap point corrections, and confidence intervals use the saved paired nested procedure.

Supplementary Table S11A Apparent threshold classifications

| **Model** | **pt** | **TP** | **FP** | **TN** | **FN** | **Se** | **Sp** |
| --- | --- | --- | --- | --- | --- | --- | --- |
| NIHSS-only | 10% | 51 | 163 | 0 | 0 | 1.000 | 0.000 |
| NIHSS-only | 20% | 36 | 121 | 42 | 15 | 0.706 | 0.258 |
| NIHSS-only | 30% | 13 | 23 | 140 | 38 | 0.255 | 0.859 |
| Primary | 10% | 49 | 162 | 1 | 2 | 0.961 | 0.006 |
| Primary | 20% | 36 | 84 | 79 | 15 | 0.706 | 0.485 |
| Primary | 30% | 20 | 28 | 135 | 31 | 0.392 | 0.828 |

Risk ≥pt is positive. Counts and Se/Sp are apparent development-sample results.

Supplementary Table S11B Apparent predictive values and likelihood ratios

| **Model** | **pt** | **PPV** | **NPV** | **LR+** | **LR−** |
| --- | --- | --- | --- | --- | --- |
| NIHSS-only | 10% | 0.238 | NE | 1.000 | NE |
| NIHSS-only | 20% | 0.229 | 0.737 | 0.951 | 1.141 |
| NIHSS-only | 30% | 0.361 | 0.787 | 1.806 | 0.868 |
| Primary | 10% | 0.232 | 0.333 | 0.967 | 6.392 |
| Primary | 20% | 0.300 | 0.840 | 1.370 | 0.607 |
| Primary | 30% | 0.417 | 0.813 | 2.283 | 0.734 |

NE indicates an undefined denominator. These ratios are apparent development-sample estimates.

Supplementary Table S11C Apparent consequences per 100 patients

| **Model** | **pt** | **Positive** | **END48 detected** | **False positive** | **END48 missed** |
| --- | --- | --- | --- | --- | --- |
| NIHSS-only | 10% | 100.00 | 23.83 | 76.17 | 0.00 |
| NIHSS-only | 20% | 73.36 | 16.82 | 56.54 | 7.01 |
| NIHSS-only | 30% | 16.82 | 6.07 | 10.75 | 17.76 |
| Primary | 10% | 98.60 | 22.90 | 75.70 | 0.93 |
| Primary | 20% | 56.07 | 16.82 | 39.25 | 7.01 |
| Primary | 30% | 22.43 | 9.35 | 13.08 | 14.49 |

Counts divided by N and multiplied by 100; false positive denotes classification, not an observed harmful intervention.

Supplementary Table S11D Optimism-corrected consequences per 100 patients

| **Model** | **pt** | **Positive** | **END48 detected** | **False positive** | **END48 missed** |
| --- | --- | --- | --- | --- | --- |
| NIHSS-only | 10% | 99.95 | 23.81 | 76.15 | 0.10 |
| NIHSS-only | 20% | 73.28 | 16.04 | 57.24 | 7.87 |
| NIHSS-only | 30% | 16.64 | 5.27 | 11.37 | 18.63 |
| Primary | 10% | 98.36 | 22.30 | 76.06 | 1.60 |
| Primary | 20% | 56.19 | 15.47 | 40.72 | 8.44 |
| Primary | 30% | 22.32 | 7.89 | 14.43 | 16.02 |

Components were corrected independently. They are estimates, not actual counts or one reconstructed confusion matrix; corrected event totals may differ slightly from observed prevalence.

Supplementary Table S11E Optimism-corrected net benefit

| **Model** | **pt** | **NB (95% CI)** |
| --- | --- | --- |
| NIHSS-only | 10% | 0.153 (0.095 to 0.222) |
| NIHSS-only | 20% | 0.017 (−0.013 to 0.120) |
| NIHSS-only | 30% | 0.004 (−0.022 to 0.066) |
| Primary | 10% | 0.139 (0.080 to 0.214) |
| Primary | 20% | 0.053 (0.006 to 0.124) |
| Primary | 30% | 0.017 (−0.015 to 0.087) |

NB is reported on the raw scale.

Supplementary Table S11F Paired incremental net benefit

| **pt** | **Primary minus NIHSS-only (95% CI)** |
| --- | --- |
| 10% | −0.015 (−0.029 to 0.005) |
| 20% | 0.036 (−0.033 to 0.068) |
| 30% | 0.013 (−0.026 to 0.073) |

Raw NB scale. Every paired ΔNB interval crosses zero. Clinical intervention benefit was not measured.

# TRIPOD+AI checklist and reproducibility

The accompanying TRIPOD_AI_CHECKLIST_FINAL_PRE_SUBMISSION.md maps all 52 checklist subitems to manuscript/supplement sections. The source is the 2024 TRIPOD+AI statement (main reference 11). Section locators are used rather than invented line numbers. The code archive records Python/R environments, source hashes, immutable prior audit, draws and checkpointed outputs. The current code sequence is documented in 10_code/README_CONSERVATIVE_V2.md. Author contributions are finalized in the main manuscript.

# Supplementary Figure S1 NIHSS spline sensitivity

Knots: 6.00, 12.00, 18.00, 22.00. Other predictors are fixed at the full-sample medians. Fitted probabilities and pointwise 95% Wald intervals are descriptive, not optimism corrected or simultaneous. Overall P=0.169; nonlinearity P=0.208. These curves do not establish biological thresholds.

# Supplementary Figure S2 Age spline sensitivity

Knots: 42.65, 57.00, 67.00, 80.35. Other predictors are fixed at the full-sample medians. Fitted probabilities and pointwise 95% Wald intervals are descriptive, not optimism corrected or simultaneous. Overall P=0.335; nonlinearity P=0.410. These curves do not establish biological thresholds.
